# The NOS/NO System in Renal Programming and Reprogramming

**DOI:** 10.3390/antiox12081629

**Published:** 2023-08-17

**Authors:** You-Lin Tain, Chien-Ning Hsu

**Affiliations:** 1 Department of Pediatrics, Kaohsiung Chang Gung Memorial Hospital, Kaohsiung 833, Taiwan; tainyl@cgmh.org.tw; 2 Institute for Translational Research in Biomedicine, Kaohsiung Chang Gung Memorial Hospital, Kaohsiung 833, Taiwan; 3 College of Medicine, Chang Gung University, Taoyuan 333, Taiwan; 4 Department of Pharmacy, Kaohsiung Chang Gung Memorial Hospital, Kaohsiung 833, Taiwan; 5 School of Pharmacy, Kaohsiung Medical University, Kaohsiung 807, Taiwan

**Keywords:** nitric oxide, kidney disease, hypertension, nitric oxide synthase, asymmetric dimethylarginine, oxidative stress, developmental origins of health and disease (DOHaD), renal programming

## Abstract

Nitric oxide (NO) is a gaseous signaling molecule with renoprotective properties. NO can be produced in NO synthase (NOS)-dependent or -independent manners. NO deficiency plays a decisive role in chronic kidney disease (CKD). Kidney development can be affected in response to adverse intrauterine conditions that induce renal programming, thereby raising the risk of developing CKD in adulthood. Conversely, detrimental programming processes could be postponed or halted prior to the onset of CKD by early treatments, namely reprogramming. The current review provides an overview of the NOS/NO research performed in the context of renal programming and reprogramming. NO deficiency has been increasingly found to interact with the different mechanisms behind renal programming, such as oxidative stress, aberrant function of the renin–angiotensin system, disturbed nutrient-sensing mechanisms, dysregulated hydrogen sulfide signaling, and gut microbiota dysbiosis. The supplementation of NOS substrates, the inhibition of asymmetric dimethylarginine (ADMA), the administration of NO donors, and the enhancement of NOS during gestation and lactation have shown beneficial effects against renal programming in preclinical studies. Although human data on maternal NO deficiency and offspring kidney disease are scarce, experimental data indicate that targeting NO could be a promising reprogramming strategy in the setting of renal programming.

## 1. Introduction

Chronic kidney disease (CKD) is a highly prevalent disease that affects 10% of the world’s population [1]. There is compelling evidence that many of the risks leading to CKD in adulthood originate in the earliest stages of life [2,3,4]. The adverse environmental events in utero that can induce negative health effects during the subsequent life of the exposed individual are now called the “developmental origins of health and disease” (DOHaD) [5]. The developing kidney is at risk of difficult intrauterine and perinatal environments, resulting in permanent functional and structural changes—that is, renal programming [6]. The kidney must adapt to any deficits in order to allow it to cope with the postnatal challenges of maintaining homeostasis from birth; therefore, this increases the susceptibility of the remaining glomeruli of developing CKD later in life [7]. Conversely, adverse programming processes could be reprogrammed ahead of the onset of CKD by early therapeutic intervention [8]. Hence, we must shift our attention from focusing only on treatment to the prevention of kidney disease in the early stages.

The kidney can be programmed by diverse early-life insults, including nutritional imbalances, placental insufficiency, maternal illnesses, exogenous stress, and exposure to alcohol, pharmaceuticals, or other toxins [2,3,4,5,6,8,9]. The putative mechanisms for these insults, which are linked to renal programming and which may promote poor renal outcomes for offspring later in life, include oxidative stress [10,11], an aberrant renin–angiotensin system (RAS) [6,12], gut microbiota dysbiosis [13], dysregulated epigenetic [4,14], impaired nutrient-sensing signals [15], and a dysregulated nitric oxide (NO) system [16,17].

NO is a lipophilic gas with multifaceted biofunctions [18]. It is known as a highly reactive radical molecule because of its unpaired electron [19]. In the kidney, NO can be produced in NO synthase (NOS)-dependent or -independent ways [20]. The three NOS isoforms are expressed in the kidney [21]. The NOS/NO system has numerous important functions in renal physiology [20,21]. Conversely, CKD is characterized by a deficiency in NO bioavailability [22]. Of note is that NO is not only vital in regulating the renal function of adults but also in the developing kidney in children [23]. As NO is a key regulator of both maternal homeostasis and fetal development during pregnancy [24], there is emerging evidence for the use of NO-related interventions as a reprogramming strategy for several adult diseases in DOHaD research [16,25,26].

This review aims to examine the current views on the NOS/NO system in the context of renal programming, with a special focus on the potential mechanisms and reprogramming strategies that target the NOS/NO pathways to avert kidney disease. We retrieved all publications that were potentially relevant to the object of study indexed in the MEDLINE, Embase, and Cochrane databases by using a variety of keywords to promote inclusiveness and sensitivity within the search. Search keywords were as follows: “kidney disease”, “developmental programming”, “DOHaD”, “nitric oxide”, “nitrite”, “nitrate”, “dimethylarginine”, “mother”, “maternal”, “pregnancy”, “gestation”, “offspring”, “progeny”, “prenatal”, “perinatal”, “reprogramming”, and “hypertension”. Also, the reference lists of the selected studies were examined for further studies that would be considered appropriate to include in this review.

## 2. The NOS/NO System in the Kidney

### 2.1. The L-arginine–NOS–NO Pathway

NO can be produced from the conversion of L-arginine to L-citrulline by the enzymatic action of NOS [18]. There are three principal NOS isoforms—neuronal NOS (nNOS), inducible NOS (iNOS), and endothelial NOS (eNOS). All the NOS isoforms are NADPH- and calmodulin-dependent, and they contain consensus binding sites. Although the three NOS isoforms are encoded by different genes, they share a 55% to 60% amino acid homology [21]. The three NOS isoforms have been localized to the kidney [21]. Splice variants of nNOS and iNOS can influence their activity and function [27,28], whereas eNOS activity is highly regulated by phosphorylation [21]. The expression of nNOS in the macula densa and eNOS in the kidney vasculature and tubules has been documented; furthermore, iNOS is expressed in these parts during pathological conditions [21].

The delivery of the substrate L-arginine and the binding of the important cofactor tetrahydrobiopterin (BH_4_) is critical for NOS activity [29]. Cationic amino acid (CAT) transporters play major roles in determining the movement of L-arginine in and out of cells. Most endogenous L-arginine production is carried out by L-citrulline conversion in the kidney [30]. As the majority of L-arginine is not used for the synthesis of NO, the relative activity of other L-arginine metabolic pathways determines the availability of L-arginine as a substrate for NOS [29]. Although the physiological concentration of L-arginine far exceeds the Km of NOS, the application of exogenous L-arginine still increases NO production, which is known as “arginine paradox” [31]. Nevertheless, NOS enzymes possess the ability to be “uncoupled” so as to produce superoxide anions instead of NO when depleted of L-arginine and BH_4_ [32].

### 2.2. The Nitrate–Nitrite–NO Pathway

Another source of NO comes from the NOS-independent pathway [33]. Nitrate (NO_3_) and nitrite (NO_2_^−^) have formerly been assumed to be fairly stable end products of NO metabolism. As both anions are eliminated mainly through urinary excretion, their sum (termed NOx) has often been utilized to estimate whole-body NOS activity [34]. On the other hand, nitrate and nitrite can be physiologically recycled to form NO in tissues and blood. Accordingly, they should be viewed as storage pools of NO, thereby complementing the NOS-dependent pathway. Dietary intake participates substantially in the pool of nitrate and nitrite in the body. Nitrates and nitrites can signal not only through the classic NO–soluble guanylate cyclase (sGC)–cGMP pathway but also through cGMP-independent mechanisms, such as nitrosylation and nitration. Unlike the NOS-dependent pathway, the nitrate–nitrite–NO pathway is oxygen-independent and potentiates during hypoxic conditions [35].

### 2.3. Endogenous NOS Inhibitor

Asymmetric and symmetric dimethylarginine (ADMA and SDMA), being structural analogs of L-arginine, act as endogenous inhibitors of NOS [36]. Protein-incorporated ADMA or SDMA is generated by protein arginine methylation. This methylation is carried out by a group of enzymes, namely protein arginine methyl transferases (PRMTs) [37]. According to specific catalytic activity, PRMT enzymes are divided into type I (PRMT1, 3, 4, 6, and 8), type II (PRMT5, 9), and type III (PRMT7) enzymes. Type I PRMTs generate ADMA, whereas type II PRMTs produce SDMA. Oxidative stress and high LDL, as occurs in CKD, upregulate PRMT1, thereby increasing ADMA production [38]. Conversely, protein arginine methylation is blocked by peptidyl arginine deiminases (PADs), which can translate methylated arginine residues to citrulline [39]. Although an arginine demethylase, JMJD6, has been identified [40], its role in the demethylation of protein-incorporated ADMA and SDMA is still unclear [41].

Free ADMA and SDMA are generated by the catabolism of proteins containing methylated arginine. Of the daily produced ADMA (~300 μmol of ADMA in adults), approximately 20% are urinary-eliminated [42]. Unlike ADMA, SDMA is generated at only half of the amount of ADMA, and the removal of SDMA only depends on renal excretion. Both ADMA and SDMA share a mutual transport system (i.e., CAT), with L-arginine for cellular transport [41]. As ADMA and SDMA have a high affinity for CATs, both may compete with L-arginine and thereby reduce its intracellular concentration. Accordingly, the application of L-arginine enables the antagonization of ADMA to solve the L-arginine paradox [31].

ADMA is metabolized by dimethylarginine dimethylaminohydrolase-1 (DDAH-1), -2 (DDAH-2), and alanine-glyoxylate aminotransferase 2 (AGXT2). DDAHs convert ADMA into dimethylamine and L-citrulline, while AGXT2 transaminates ADMA to form α-keto-δ-(N^G^, N^G^-dimethylguanidino) valeric acid (DMGV) [43]. The expression and/or activity of DDAHs can be inhibited to increase ADMA by oxidative stress [44], hyperglycemia [45], and angiotensin II administration [46]. DDAH1, DDAH2, and AGXT2 are all expressed in the kidney [47,48]. Unlike DDAHs, AGXT2 not only metabolizes ADMA but also SDMA [48]. Only one study has revealed that D-β-aminoisobutyric acid inhibits the metabolism of ADMA and SDMA when induced by AGXT2 [48], but the detailed mechanism through which this occurs remains to be further evaluated. Apart from dimethylarginines, another methylarginine residue—N^G^ monomethyl-l-arginine (NMMA)—is also produced in mammals and acts as a NOS inhibitor [47]. Given that its blood level is much lower than those of ADMA and SDMA, data are scarce regarding its pathophysiological role in clinical conditions.

**Figure 1:**
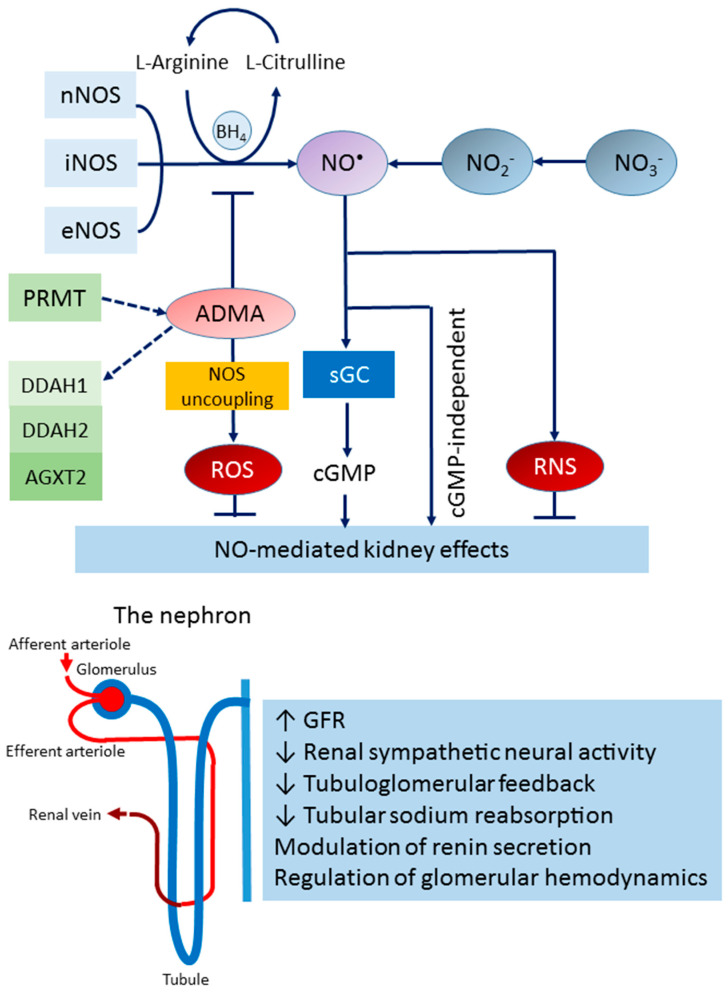
The NOS/NO system and the potential effects of NO on kidney functions: NO is generated from the NOS-dependent or -independent pathways. NO bioactivity has been associated with several effects in kidney function, mainly via cGMP-dependent mechanisms, although cGMP-independent mechanisms have also been reported. NOS can be inhibited by asymmetric dimethylarginine (ADMA). ADMA is generated by protein arginine methyl transferases (PRMTs). Dimethylarginine dimethylaminohydrolase-1 (DDAH-1) and -2 (DDAH-2), and alanine-glyoxylate aminotransferase 2 (AGXT2) can metabolize ADMA. NOS uncoupling contributes to reactive oxygen species (ROS) generation. Both ROS and reactive nitrogen species (RNS) can inhibit NO-mediated kidney effects.

The blood concentrations in the ADMA, SDMA, and NMMA of healthy humans are approximately 0.4 µM, 0.4 µM, and 0.1 µM, respectively [49]. Free ADMA is a potent (IC50, 1.5 µM) nNOS inhibitor but a relatively weak (IC50, 12 µM) eNOS inhibitor [50]. At a molar basis (1 µM), the order of the inhibitory potency toward nNOS activity is around 5: 4: 1 for ADMA: NMMA: SDMA [50]. Accordingly, compared with ADMA, NMMA and SDMA are less potent nNOS inhibitors. The biochemical pathways that participate in the NOS/NO system are illustrated in Figure 1.

### 2.4. NO in Kidney Health and Disease

In the kidney, NO maintains many significant processes, including the autoregulation of renal hemodynamics, the modulation of renal sympathetic neural activity, the modulation of medullary blood flow, the mediation of pressure natriuresis, the modulation of sodium transport, the blunting of tubuloglomerular feedback, the release of renin, and the inhibition of tubular sodium reabsorption [20,21,51,52]. From an integrative point of view, the inhibition of the NOS/NO system impairs these homeostatic mechanisms and damages renal health.

Take CKD for example; several lines of evidence indicate deficiency in NO plays a decisive role [22]. First, reduced L-arginine availability could stem from insufficient dietary supplementation or via reduced de novo citrulline-to-arginine conversion (which occurs in patients with CKD [53]). Another line of evidence comes from NOS inhibitors. Data from systemic reviews support the notion that high circulating ADMA and SDMA levels are risk factors for CKD and cardiovascular morbidity [54]. Third are the studies of NOS knockout mice. Mice without nNOS are defective in the absorption of fluid and bicarbonate in the proximal tubule, and thus develop metabolic acidosis [55]. A deficit in eNOS-generated NO exacerbates the kidney damage in several CKD models [56,57]. Fourth, renal nNOS protein levels and activity decrease with kidney injury, which is associated with low NO production and hypertension in several rat models of CKD [22,58,59]. Moreover, processes apart from NO production could also reduce NO bioavailability. Oxidative stress could diminish the bioactivity of NO by inhibiting DDAH activity to increase ADMA, thus oxidizing cofactor BH4 to uncouple NOS, limiting the access of NO to target tissues and scavenging NO by superoxide to produce peroxynitrite [60]. As a result, the oxidatively inactivated NO system in the kidney might promote CKD and hypertension [61]. Given that the NOS/NO system is impaired in CKD, a novel strategy should target NO for the prevention rather than just the treatment of kidney disease.

## 3. The NOS/NO System in Renal Programming

### 3.1. NO in Pregnancy

During pregnancy, NO is implicated in the implantation and regulation of fetoplacental vascular reactivity, trophoblast invasion and apoptosis, placental angiogenesis, and fetal development [62]. In pregnant mice, all three NOS isoforms are present in uterine tissue from the 4th to 8th gestational days, indicating the role of NO in the mechanisms of implantation [63]. In early gestation, low ADMA and the concomitantly high NO are responsible for hemodynamic adaptation and uterine relaxation, and this occurs in order to maintain normal fetal growth. Conversely, physiologically high ADMA in late pregnancy can antagonize the NO-induced uterine relaxation to increase uterine contractile activity for successful delivery.

In humans, the plasma concentrations of nitrate/nitrite are raised in normal pregnancy [64], while deficiency in NO and high ADMA seem to be involved in compromised pregnancy [65]. Maternal plasma L-arginine level is lower in pregnancies that are complicated by intrauterine growth retardation (IUGR) [66]. In addition, the plasma concentration of L-arginine and placental eNOS protein levels are lower in preeclamptic compared with healthy pregnant women [67]. ADMA and SDMA concentrations were stated to rise in preeclamptic pregnancies [68,69]. The evidence from animal models has revealed reduced L-arginine levels, decreased NO, and increased superoxide formation, thereby resulting in the excessive formation of peroxynitrite in preeclampsia placentas [70].

Taken together, these findings show that the complex regulation of different components in the NOS/NO system is essential for ensuring a successful pregnancy and fetal development.

### 3.2. NO in Renal Programming

The basic functional unit of the kidney is the nephron. In humans, the total number of nephrons is about 1,000,000 in each kidney and varies over a 10-fold range [71]. In humans, nephrogenesis initiates at week 3 and ends at 36 weeks of gestation [72]. In rodents, kidney development lasts after birth and stops at 1–2 weeks postnatally [73]. Branching morphogenesis is an important determinant of the final nephron number of the kidney [74]. Early-life environmental risk factors can impair kidney development, reduce nephron number, and cause a wide range of malformed kidneys, i.e., congenital anomalies of the kidney and urinary tract (CAKUT) [75]. A low nephron number in renal programming may enact a first hit to the kidneys, which makes the remaining nephrons more vulnerable to developing CKD in the face of second-hit kidney injuries [7]. A kidney endowed with fewer nephrons may be less able to adapt, leading to glomerular hyperfiltration, compensatory glomerular hypertrophy, and further reductions in nephrons [76].

To date, the impact of an early-life-impaired NOS/NO system on the development of kidney disease in humans remains largely unknown. Epidemiologic studies have found an increased risk for kidney disease in later life concerning low birth weight (LBW) and prematurity [2,77]; notably, both are reliable as a surrogate for nephron endowment [72]. A previous meta-analysis encompassing over 2 million subjects concluded that individuals who had an LBW have a 70% increased risk in the development of CKD [78]. A case–control study with more than 2000 children with CKD indicated that an LBW, maternal obesity, and maternal gestational diabetes impact the risk of CKD [79]. Another case–control study that recruited over 1.6 million infants concluded that several risk factors for CAKUT are related to the NO system, and this covers prematurity, maternal gestational diabetes, LBW, and maternal thalassemia [65,80].

Currently, the number of nephrons can only be determined in vitro. Renal biopsy in children is considered technically more difficult than in adults, especially in the fetus and neonates. In humans, a direct link between low nephron number and CKD later in life remains to be evaluated further. This is the reason much of our knowledge regarding the molecular mechanisms underlying NO-related renal programming and reprogramming interventions that are used to target NO in preventing kidney disease mostly stem from preclinical studies.

In a maternal NO deficiency rat model, mother rats that are treated with N^G^-nitro-L-arginine-methyl ester (L-NAME, a NOS inhibitor) during gestation incurred renal programming and offspring hypertension [81]. A deficient amount of NO significantly altered the renal transcriptome in neonatal kidneys, resulting in 2289 differentially expressed genes (DEGs; 1259 up- and 1030 downregulated). Among them, multiple genes were associated with kidney development and epigenetic regulation. In addition, there are 22 Kyoto Encyclopedia of Genes and Genomes (KEGG) pathways that are enriched statistically between L-NAME-treated offspring rats and control rats. Among them, the renin–angiotensin system (RAS) [82], arachidonic acid metabolism pathway [83], aldosterone-regulated sodium reabsorption [84], and PPAR signaling pathway [85] are putative mechanisms that underly renal programing and which are linked to the development of kidney disease.

Another study revealed that ADMA impairs branching morphogenesis and decreases nephron number [86]. Embryonic kidneys grown in 2 or 10 µM of ADMA contained fewer nephrons in a dose-dependent manner [86]. A total of 1221 DEGs in cultured fetal kidneys treated with 10 µM of ADMA [48] were identified by next-generation sequencing (NGS) analysis. Among them, *Avpr1a*, *Hba2*, *Ephx2*, *Hba-a2*, and *Npy1r* were found to be connected to offspring hypertension in models of renal programming [87,88]. Hence, the results from these studies proposed a connection between an impaired NOS/NO system and renal programming during gestation; thus, this finding could represent a strong contribution to understanding offspring kidney disease.

### 3.3. Animal Models of Renal Programming Linked to Impaired NOS/NO System

Animal models have been indispensable in elucidating the potential causative mechanisms underlying renal programming to assess whether certain early-life risk factors at specific windows of development influence offspring outcomes. Particularly, animal models have provided more direct insight into the interconnection between the NOS/NO system and renal programming. Table 1 summarizes the studies that documented animal models of renal programming linked to NO that were only restricted to adverse environmental stimuli starting in the gestation and/or lactation period; as such, these studies covered the periods of nephrogenesis [81,83,86,89,90,91,92,93,94,95,96,97,98,99,100,101,102,103,104,105].

Table 1 illustrates that maternal and fetal exposure to a range of environmental insults may impair nephrogenesis, result in morphological and functional changes, and cause adverse renal outcomes. Studies have applied various nutrients or diets to determine their impact on renal programming. A variety of nutritional insults can be clustered into diverse animal models that aim to limit calorie intake [90,91,92], limit protein intake [98], induce the insufficient intake of zinc [89] or iron [103], and increase the feeding of a diet with a high level of fructose [83,95] or fat [101,102]. Another aspect interfering with renal programming is maternal or fetal exposure to illness, such as diabetes [86], preeclampsia [81,93,94], CKD [97], and solitary kidneys [104]. Also, renal programming can be induced by the maternal administration of glucocorticoids [99,105], ADMA [96], or TMAO [96]. Rat models have been extensively used in studying renal programming. Given that every month for the animal is approximately equivalent to 3 human years [74], Table 1 presents the age at measure that allows certain calculations to refer to human ages.

Several NOS/NO-associated mechanisms participate in renal programming, including reduced L-arginine concentration [83,95,96,101], reduced L-arginine-to-ADMA ratios [81,84,89,90,91,93,94,95,99], reduced renal eNOS protein levels [98,100], reduced renal NOS activity [89], reduced urinary NOx levels [90,98,104], reduced urinary cGMP levels [81,94], reduced renal NO production [103], and increased ADMA [83,86,91,92,93,95,97,99,102] and SDMA [102]. These observations support the notion that NO deficiency is attributed to multiple mechanisms and is involved in the pathogenesis of renal programming.

In regard to renal programming, it should be noted that a diverse range of phenotypes has been reported, including reduced nephron number [87], glomerular hypertrophy [89,91,92,103], elevated blood pressure [81,86,91,92,93,94,95,96,97,98,99,100,101,103,104,105], reduced glomerular filtration rate (GFR) [90,104], tubulointerstitial injury [86,91,92], increased renal NHE3 protein levels [81,94], altered renal transcriptomes [81,94,100], increased plasma Cr concentration [94,100], and renal hypertrophy [97,99].

## 4. Reprogramming Strategies Targeting the NOS/NO System

Several interventions targeting the NOS/NO system acting as a reprogramming strategy to counteract programming processes have been applied in various animal models of renal programming, some of which are listed in Table 2 [84,89,90,92,93,97,99,105,106,107,108,109,110,111,112].

There are several ways to improve NO bioavailability—by supplementation of NOS substrate, by inhibition of ADMA, by administration of NO donors or nitrodilators, and by enhancement of NOS. Table 2 presents several such studies in which reprogramming interventions were applied during gestation and lactation.

### 4.1. NOS Substrates

L-arginine supplementation has been utilized to produce NO in experimental studies [115]. However, the use of L-arginine was not applied during pregnancy and lactation in most studies, thereby limiting our understanding of its reprogramming effect. Only one study shows that the maternal administration of L-arginine and antioxidants can protect fawn-hooded hypertensive rats (FHH) against hypertension, proteinuria, and glomerulosclerosis in adulthood [107]. Of note is that L-arginine is not an ideal NO precursor, as it has multiple metabolic fates. L-citrulline can act as an L-arginine precursor for NO synthesis. L-citrulline is more bioavailable than L-arginine because of its bypass of liver metabolism, and it can be converted back to L-arginine in the kidney [116]. Maternal L-citrulline supplementation can protect adult offspring against hypertension that is caused via developmental origins, which are induced by streptozotocin-induced maternal diabetes [86], maternal caloric restriction [91], maternal L-NAME administration [94], and prenatal dexamethasone exposure [99]. In SHRs, maternal supplementation with L-citrulline enhanced renal NO and blocked the development of hypertension [108]. Whether maternal NO precursor supplementation increases renal NO and thereby prevents renal programming remains to be further investigated.

### 4.2. ADMA-Lowering Agents

Several commonly used prescription drugs have shown ADMA-lowering effects in human and animal studies, as has been reviewed elsewhere [35,117]. Resveratrol, melatonin, and N-acetylcysteine can reduce ADMA by augmenting the expression/activity of DDAHs.

Maternal treatment with resveratrol, melatonin, garlic oil, or N-acetylcysteine has been shown to induce ADMA-lowering action, which averts offspring hypertension in a maternal caloric restriction model [92], prenatal dexamethasone plus TCDD exposure model [109], a maternal CKD model [110], and a prenatal dexamethasone plus postnatal high-fat-diet model [111], respectively. Additionally, maternal dimethyl fumarate treatment, a known activator of nuclear factor erythroid-derived 2-related factor 2 (Nrf2), prevents two-hit-induced programmed hypertension in male offspring, which has been associated with a reduction in ADMA [112]. Moreover, maternal butyrate supplementation reduced ADMA and prevented offspring hypertensions induced by concurrent maternal high-fructose consumption [95]. Another study showed that garlic oil supplementation during pregnancy and lactation prevented maternal CKD-induced offspring hypertension and that it was also relevant to a reduction in ADMA [108]. Whether they inhibit ADMA-producing enzymes or enhance ADMA-metabolizing enzymes remains to be further evaluated.

It is worthwhile to note that several ADMA-lowering agents, such as resveratrol, melatonin, NAC, and Nrf2, are tightly connected to oxidative stress. Their beneficial actions might counteract renal programming processes in different ways other than just targeting the ADMA/NO pathway. Unfortunately, a specific ADMA-lowering agent remains inaccessible in clinical practice. The discovery of specific PRMT inhibitors and DDAH agonists might have clinical benefits in terms of lowering ADMA and improving NO availability.

### 4.3. NO Donors and Nitrodilators

Despite recent advances in the development of NO donors [118], their roles in renal programming are scarcely known. In our recent work, we presented the antihypertensive effect of a NO donor, i.e., diethylenetriamine/NO adduct (DETA NONOate), in young rats with CKD [119]. In addition to the NO donor, a few of the nitrodilators in renal programming were examined. Nitrodilators, such as nitroglycerin, pentaerythritol tetranitrate (PETN), and molsidomine, possess the capacity for NO-mimetic vasodilatory actions via releasing NO from sources other than their own molecules [120,121]. PETN and molsidomine, both nitrodilators, have been found to provide benefits against hypertension in SHRs and FFH rats, respectively [113,114].

### 4.4. Others

Our prior work showed that the dietary supplementation of nitrate, the most commonly used NO precursor, enabled the prevention of hypertension development in young SHRs [122]; however, its reprogramming effect remains to be further investigated. One study revealed that melinjo (Gnetum gnemon) seed extract supplementation during lactation could augment eNOS expression and protect maternal high-fructose-diet-induced hypertension in adult female offspring [101]. As NOS activity can be regulated in a variety of ways, there might be other approaches through which to improve NO bioavailability via enhancing NOS activity. For example, drugs interfering with the RAS and statins were reported to restore NOS activity by reducing oxidative stress [31]. Targeting NOS modification might also be an interesting alternative strategy to explore.

## 5. Mechanisms of the Renal Programming Linked to the NOS/NO System

**Figure 2:**
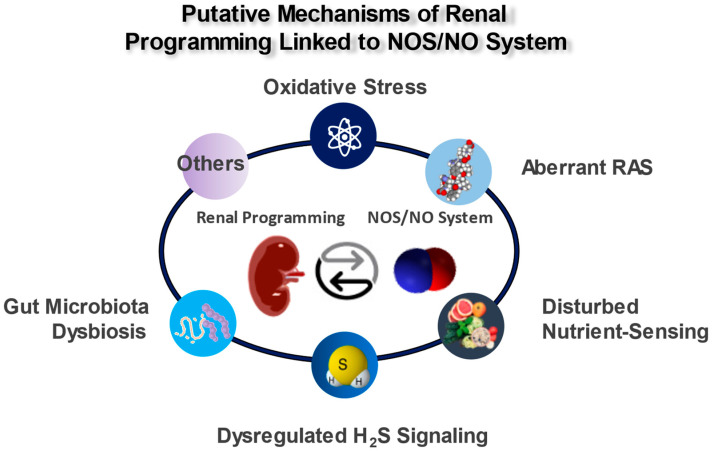
Schema outlining the putative mechanisms of the renal programming linked to the NOS/NO system.

Considering the multifaceted roles of NO, there might be an interaction between NO and other mechanisms that are behind renal programming and which participate in the pathogenesis of kidney disease in later life. Animal models have shed light on several putative mechanisms, covering oxidative stress, alterations of the RAS, disturbed nutrient-sensing mechanisms, dysregulated hydrogen sulfide (H_2_S) signaling, and gut microbiota dysbiosis. All of these observations illustrate a significant correlation between the impaired NOS/NO system and other mechanisms that participate in renal programming (Figure 2); each of which is discussed in turn.

### 5.1. Oxidative Stress

The developing fetus, with its decreased antioxidant defense, is susceptible to oxidant injury [11]. Oxidative stress could lead to NOS uncoupling, thus diminishing NO formation. NO reacts rapidly with superoxide to form the potent oxidant peroxynitrite, thus resulting in nitrative stress [58]. Additionally, ADMA is not just a NOS inhibitor but also an ROS inducer. Hence, renal programming is possibly the result of interactions between NO deficiency and oxidative stress. Growing evidence shows that an impaired NOS/NO system and oxidative stress simultaneously exist in a variety of models of renal programming. In a maternal caloric restriction model, 8-hydroxydeoxyguanosine (8-OHdG)—a marker of oxidant-induced DNA damage [123]—is increased in the offspring’s kidney, with an increase in ADMA and a decrease in AAR [91,92]. In a model of maternal L-NAME administration [81], the renal concentrations of F2-isoprostane, a marker of lipid peroxidation, were increased. Likewise, several models of renal programming (listed in Table 1), such as a maternal high-fructose diet [83], prenatal dexamethasone exposure [99], and a maternal high-fat diet [100], have given rise to NO deficiencies that coincide with enhanced renal 8-OHdG expression. Together, these data point to a specific interconnection between oxidative stress and an impaired NOS/NO system in renal programming.

### 5.2. Aberrant RAS

The aberrant activation of the RAS is known to have a role in renal programming [12]. The RAS genes are highly expressed in the developing kidney [124], while their mutations are related to kidney malformation [125]. The main components of the classic RAS include angiotensin-converting enzymes (ACEs), angiotensin (ANG) II, and angiotensin II type 1 receptor (AT1R). Under pathophysiological conditions, the classic RAS can be activated to induce renal inflammation, activate NAD(P)H oxidase to increase oxidative stress, and stimulate the release of cytokine/chemokines—all contributing to kidney damage [126].

The ANG II activation of AT1R leads to oxidative stress and a reduced NO bioavailability, while NO can counterbalance the vasoconstrictive effect of ANG II [127]. In a maternal L-NAME exposure model, deficiency in NO resulted in offspring hypertension, which coincided with an increased mRNA expression of renin and ACE in the offspring’s kidneys [81]. Similarly, a NO deficiency in prenatal dexamethasone-induced hypertension is relevant to increased classic RAS genes and AT1R expressions in the kidneys of the offspring [99]. In another model of renal programming, the blockade of the RAS by aliskiren protected adult rat offspring against hypertension that was programmed by maternal caloric restriction [128]. The protective action of aliskiren was not merely directed upon the RAS but also through decreases in plasma ADMA levels and increases in urinary NOx levels [128]. Another example is a perinatal high-fat model. The protective actions of the maternal Nrf2 activator cover not only a decrease in plasma ADMA but also the downregulation of several classic RAS genes (i.e., renin, ACE, and AT1R) [112].

As there is an apparent role for the balance between the RAS and NO system in the pathogenesis of renal programming, there exists a rising need to better understand the early targeting of the RAS, as it can restore the NO system such that it can prevent renal programming and adult kidney disease.

### 5.3. Disturbed Nutrient Sensing

Nutrient-sensing mechanisms in pregnancy that respond to specific nutrients ensure that maternal metabolism functions and fetal growth rate coordinate properly [129]. In contrast, disturbed nutrient-sensing mechanisms lead to adverse fetal programming and have an apparent role in renal programming [130].

AMP-activated protein kinase (AMPK) is activated by falling cellular energy [131]. As reviewed elsewhere, newly discovered evidence shows that a disturbed AMPK signaling pathway is linked to the developmental programming of kidney disease, while early-life AMPK activation can aid in preventing renal programming-induced disorders [132]. AMPK can phosphorylate eNOS at serine 1177, which is involved in the enhancement of NOS activity [133]. Resveratrol, an AMPK activator, prevents oxidative NO inactivation via AMPK activation, thereby enhancing NO bioavailability [134]. In line with this observation, resveratrol has been found to avert high-fat-diet-induced hypertension, which is associated with increased renal AMPK2α expression, decreased renal ADMA concentration, and reduced oxidative stress damage [135].

Another key nutrient-sensing signal is the peroxisome proliferator-activated receptor (PPAR) [85]. Several PPAR target genes participate in renal programming [85,136], including iNOS and eNOS. In addition, the PPAR signaling pathway is recognized as a significantly regulated KEGG pathway in several models of renal programming; these models cover maternal caloric restriction, maternal diabetes, and a maternal high-fructose diet [88]. Worthy of note are the three models listed in Table 1 as NO-related renal programming. These results offer evidence for the interplay between disrupted nutrient-sensing mechanisms and the impaired NOS/NO systems that are implicated in renal programming.

### 5.4. Dysregulated H_2_S Signaling

Similar to NO, H_2_S has an apparent role in renal physiology and the regulation of BP [137]. They both share biological targets and enable a chemical interaction with each other [138]. There exists an H_2_S/NO crosstalk via S-nitrosylation and S-sulfhydration for the same protein-incorporated cysteine residues, hence allowing H_2_S and NO to regulate each other [138]. H_2_S could provide a backup system for the vasorelaxation that becomes important when NO is reduced. One example of this is the inhibition of NO by L-NAME in rats, where the development of hypertension can be prevented by the administration of sodium hydrosulfide (NaHS, a H_2_S donor) via the restoration of NO bioavailability [139]. Though H_2_S and NO share the same soluble guanylyl cyclase (sGC)–cyclic guanosine monophosphate (cGMP) pathway that induces vasorelaxation, they act at different levels, with H_2_S inhibiting cGMP degradation and NO increasing cGMP through the stimulation of sGC [138]. As the crosstalk between NO and H_2_S is particularly complex, and as detailed reviews are beyond the scope of this paper, readers are referred elsewhere [138,140,141].

A growing body of evidence supports the notion that dysregulated H_2_S signaling is implicated in the developmental programming of adult diseases [142]. Although the protective role of NAC against the renal programming that is induced by prenatal dexamethasone exposure plus postnatal high-fat diets was linked to reducing ADMA [111], it also caused an upregulation in the gene expression of H_2_S-producing enzymes. Another study revealed that supplementing garlic oil in gestation and lactation prevented maternal high-fat-diet-induced offspring hypertension, which not only coincided with the restoration of NO bioavailability but also enhanced the expression and activity of H_2_S-generating enzymes in the kidneys of the offspring [110]. Worthy of note is that both NAC and garlic oil are precursors of H_2_S, and they are used as H_2_S-based reprogramming strategies for DOHaD-related diseases [142]. Considering the complex interplay between H_2_S and NO, the reprogramming effect responding to interventions based on each one—either alone or in combination—is interesting and should be further evaluated.

### 5.5. Gut Microbiota Dysbiosis

Alterations in gut microbial composition and their metabolites are related to CKD [143]. Maternal gut microbiota can impact their offspring’s gut microbiota, which brings attention to the importance of maternal insults in the adverse impact on offspring gut microbiota later in life [144,145].

Several models of renal programming have been conducted to assess gut microbiota in the developmental programming of kidney disease, whereby a maternal high-fructose diet [95], combined TMAO plus ADMA exposure [96], maternal CKD [110], and a perinatal high-fat diet are covered [146]. As mentioned in Table 1, all these models are relevant to impaired NOS/NO systems.

Short-chain fatty acids (SCFAs) are major microbial metabolites [147]. Dysregulated SCFAs and their receptors are connected to renal programming [13]. Conversely, SCFA supplementation during pregnancy and lactation has been used as a gut-microbiota-targeted reprogramming intervention to prevent the developmental programming of adult disease [148]. Butyrate, an example of a dominant SCFA, has been reported to enhance NO production [149]. In CKD, butyrate-generating microbes and butyrate production declined with disease severity [150]. In a maternal high-fructose-diet model [95], offspring hypertension was associated with a high ADMA level and a low plasma AAR, as well as alterations in gut microbial composition. Maternal butyrate supplementation not only restores the NO system but also increases plasma SCFA concentrations, which is a finding worthy of further evaluation with respect to the interaction between the NO system and gut microbiota.

## 6. Conclusions

Many studies have indicated the involvement of the NOS/NO system in kidney physiology and the pathological processes of kidney disease. Our review extends the scope of the NOS/NO system in relation to renal programming and reprogramming so as to provide an innovative strategy in preventing CKD and for advancing global kidney health. Growing evidence shows the pathogenic role of an impaired NOS/NO system in renal programming and in the development of adult kidney disease. Numerous NO-related reprogramming interventions are associated with improved kidney health in preclinical studies. However, there is still little information about the interaction between the NOS/NO system and the other putative mechanisms that are behind renal programming. Additional research is required to determine the optimal dosage and duration of NO-based interventions in the different models of renal programming before clinical translation. Whether these NO-based treatments in pregnancy and lactation add benefit to the ongoing search for the prevention of adult kidney disease in humans is worth investigating in specifically designed trials using pregnant women with risk factors for renal programming.

## Figures and Tables

**Table 1:** Animal models of NO-associated renal programming.

Animal Models	Species/ Gender	Age at Measure (Weeks)	NOS/NO System	Renal Programming	Ref.
Maternal zinc deficiency during gestation and lactation	Wistar rat/M and F	3	↓ Renal NOS activity	↓ Nephron number and glomerular hypertrophy	[89]
Maternal caloric restriction during gestation and lactation	SD rat/M and F	8	↓ Urinary NOx level	↑ BP and ↓ GFR	[90]
Maternal caloric restriction during gestation and lactation	SD rat/M	12	↑ ADMA, ↓ AAR	↓ Nephron number, ↑ tubulointerstitial injury, ↑ BP and glomerular hypertrophy,	[91,92]
Streptozotocin-induced maternal diabetes	SD rat/M	12	↑ ADMA, ↓ AAR	↓ nephron number, ↑ tubulointerstitial injury and ↑ BP	[86]
Maternal suramin exposure during gestation and lactation	SD rat/M	12	↑ ADMA, ↓ AAR	↑ BP	[93]
Maternal L-NAME administration during gestation and lactation	SD rat/M	12	↓ Renal NO, ↓ urinary cGMP level	↑BP, ↑ renal NHE3 protein level, and altered renal transcriptome	[81,94]
Maternal high-fructose diet during gestation and lactation	SD rat/M	12	↑ ADMA, ↓ L-arginine, and ↓ AAR	↑ BP and altered renal transcriptome	[83,95]
Maternal ADMA administration in gestation	SD rat/M	12	↓ L-arginine and ↓ AAR	↑ BP	[96]
Maternal TMAO administration in gestation	SD rat/M	12	↓ L-arginine	↑ BP and ↑ plasma Cr concentration	[96]
Maternal CKD	SD rat/M	12	↑ ADMA, ↓ AAR	↑ BP and renal hypertrophy	[97]
Maternal low-protein diet during gestation and lactation	Wistar rat/M	14	↓ Renal eNOS phosphorylated protein level and ↓ urinary NOx level	↑ BP	[98]
Prenatal dexamethasone administration at gestational days 15 and 16	SD rat/M	16	↑ ADMA, ↓ renal NO	↑ BP and renal hypertrophy	[99]
Maternal high-fat diet during gestation and lactation	SD rat/M	16	↓ L-arginine and ↓ AAR	↑ BP	[100]
Maternal high-fructose diet in gestation	Wistar rat/F	17	↓ Renal eNOS protein level	↑ BP	[101]
Maternal high-fat diet during gestation and lactation	SD rat/M	24	↑ ADMA and ↑ SDMA	↑ Plasma Cr concentration	[102]
Maternal iron deficiency diet in pregnancy	SD rat/M	24	↓ Renal NO	↑ BP, ↑ renal collagen deposition, and glomerular hypertrophy	[103]
Fetal unilateral nephrectomy model	Sheep/M and F	24	↓ Urinary NOx level	↑ BP and ↓ GFR	[104]
Prenatal betamethasone exposure at gestational days 80 and 81	Sheep/M and F	72	↓ NO	↑ BP	[105]

Studies that were tabulated based on animal species and age at evaluation. SD = Sprague–Dawley; cGMP = cyclic guanosine monophosphate; ADMA = asymmetric dimethylarginine; SDMA = symmetric dimethylarginine; NOx = nitrite + nitrate; Cr = creatinine; NHE3 = type 3 sodium hydrogen exchanger; M = male; F = female; NO = nitric oxide; BP = blood pressure; and GFR = glomerular filtration rate; ↑ = increase; ↓ = decrease.

**Table 2:** Reprogramming strategies targeting the NOS/NO system in animal models of renal programming.

Interventions	Animal Models	Species/ Gender	Age at Measure (Weeks)	Protective Effect	Ref.
L-arginine + antioxidants	Genetic hypertension	FHH/M and F	36	Prevented high BP, proteinuria, and glomerulosclerosis	[107]
L-citrulline	Streptozotocin-induced maternal diabetes	SD rat/M	12	Prevented kidney damage and high BP and protected against reduced nephron number	[86]
L-citrulline	Maternal caloric restriction	SD rat/M	12	Prevented kidney damage and protected against reduced nephron number	[91]
L-citrulline	Maternal L-NAME administration	SD rat/M	12	Prevented high BP	[94]
L-citrulline	Prenatal dexamethasone exposure	SD rat/M	12	Prevented high BP and protected against reduced nephron number	[99]
L-citrulline	Genetic hypertension	SHR/M and F	50	Prevented high BP	[108]
Melatonin	Maternal caloric restriction	SD rat/M	12	Prevented high BP	[92]
Resveratrol	Prenatal dexamethasone plus TCDD exposure	SD rat/M	12	Prevented high BP	[109]
Garlic oil	Maternal CKD	SD rat/M	12	Prevented high BP	[110]
Butyrate	Maternal high-fructose diet	SD rat/M	12	Prevented high BP	[95]
N-acetylcysteine	Prenatal dexamethasone plus postnatal high-fat diet	SD rat/M	16	Prevented high BP	[111]
Dimethyl fumarate	Prenatal dexamethasone plus postnatal high-fat diet	SD rat/M	16	Prevented high BP	[112]
Pentaerythritol tetranitrate	Genetic hypertension	SHR/M and F	24	Prevented high BP	[113]
Molsidomine	Genetic hypertension	FHH/M and F	42	Prevented high BP	[114]
Melinjo (Gnetum gnemon) seed extract	Maternal high-fructose diet	Wistar rat/F	17	Prevented high BP	[101]

Studies tabulated based on the types of reprogramming intervention and animal models. SD = Sprague–Dawley; M = male; F = female; and BP = blood pressure.
